# Effect of small scale transport processes on phytoplankton distribution in coastal seas

**DOI:** 10.1038/s41598-018-26857-9

**Published:** 2018-06-05

**Authors:** Ismael Hernández-Carrasco, Alejandro Orfila, Vincent Rossi, Veronique Garçon

**Affiliations:** 1 Balearic Islands Coastal Observing System, ICTS-SOCIB, Parc Bit, Edificio Naorte, 2nd floor, 07121 Palma de Mallorca, Spain; 2 Oceanography and Global Change Department, IMEDEA (CSIC-UIB), 07190 Esporles, Spain; 3 Mediterranean Institute of Oceanography (UM 110, UMR 7294), CNRS, Aix Marseille Univ., Univ. Toulon, IRD, 13288 Marseille, France; 4 LEGOS, Laboratoire d’Etudes en Géophysique et Océanographie Spatiales, CNRS, 18, Avenue Edouard Belin, 31401 Toulouse Cedex 9, France

## Abstract

Coastal ocean ecosystems are major contributors to the global biogeochemical cycles and biological productivity. Physical factors induced by the turbulent flow play a crucial role in regulating marine ecosystems. However, while large-scale open-ocean dynamics is well described by geostrophy, the role of multiscale transport processes in coastal regions is still poorly understood due to the lack of continuous high-resolution observations. Here, the influence of small-scale dynamics (O(3.5–25) km, i.e. spanning upper submesoscale and mesoscale processes) on surface phytoplankton derived from satellite chlorophyll-a (Chl-a) is studied using Lagrangian metrics computed from High-Frequency Radar currents. The combination of complementary Lagrangian diagnostics, including the Lagrangian divergence along fluid trajectories, provides an improved description of the 3D flow geometry which facilitates the interpretation of two non-exclusive physical mechanisms affecting phytoplankton dynamics and patchiness. Attracting small-scale fronts, unveiled by backwards Lagrangian Coherent Structures, are associated to negative divergence where particles and Chl-a standing stocks cluster. Filaments of positive divergence, representing large accumulated upward vertical velocities and suggesting accrued injection of subsurface nutrients, match areas with large Chl-a concentrations. Our findings demonstrate that an accurate characterization of small-scale transport processes is necessary to comprehend bio-physical interactions in coastal seas.

## Introduction

Coastal marine ecosystems have received increasing attention in the last decades due to their important contribution to the global carbon budget^1^, to the world ocean’s primary production^2^ and to withdraw the global fisheries^3^. The hydrodynamics in coastal areas is characterized by a complex interaction of multiscale processes where the energetic inputs from the atmosphere balance with the dissipation at the coast and at the seabed, resulting in specific dynamical features whose spatial and temporal characteristics cover a wide range of scales which differ from those of the open ocean^4,5^.

These multiscale dynamical processes have profound consequences on the transport and dispersion of biogeochemical tracers, whose knowledge is crucial to understand the mechanisms regulating marine ecosystem (see Martin, 2002^6^ and Mahadevan^7^ for reviews). In general, fluxes of nutrients into the depleted surface layers of both open and coastal oceans depend on the occurrence and magnitude of vertical motions as well as on horizontal advection patterns and small-scale filaments from distant nutrient-rich regions^6–8^. In oligotrophic regions, mesoscale dynamical structures have been related to chlorophyll distribution^9^, sea bird foraging behavior^10^ and vertical transport^11^. In eutrophic regions, the role of mesoscale turbulence on surface phytoplankton was studied from satellite-derived currents^12^ and based on model outputs^13^. Contrarily to what is expected in the open ocean, both latter studies documented an anti-correlation between mixing and chlorophyll-a biomass in eastern-boundary upwelling zones. The overall effect of mesoscale eddies reducing near-shore productivity in upwelling systems was corroborated by Gruber *et al*.^14^, using satellite data together with coupled simulations. However, little is known about bio-physical interactions within coastal regions not forced by upwelling-favourable trade winds.

Recents studies using drifter observations^15–17^ and numerical models^18–22^ have shown that submesoscale processes play an important role on the dispersal and transport of tracers. Submesoscale refers to length-scales that are lower than the first baroclinic deformation radius, *R*_*d*_, ranging from 0.1 km to 10 km^23^. In general, the relative dispersion rates at the submesoscale, locally driven by small-scale structures, are significantly higher than for mesoscale dynamics^17^. Berta *et al*.^24^ showed that submesoscale flow structures are crucial to determine tracer patch deformation and Haza *et al*.^20^, observed that submesoscale features could produce a leakage of particles from the mesoscale eddies. However the submesoscale effects on transport processes are still only partially understood^16,25^, especially for active tracers and in complex and turbulent coastal flows^26^.

The lack of high resolution observations of ocean currents from observations impeded the study of the high spatio-temporal variability of coastal dynamics. This issue has been partially addressed in the last years by the development of High-Frequency Radar (HFR) systems which provide velocity measurements of the surface coastal ocean^27–29^. The use of HFR data has the potential to provide information with the necessary resolution in both space and time to unravel small scale bio-physical interactions.

We study here the transport processes in a coastal region monitored by HFR in the Ibiza channel, a key site for water circulation in the Western Mediterranean basin (see Fig. S1 in SI). It is indeed one of the main pathways for the north-south exchanges of surface and intermediate waters between the Balearic sea and the Algerian Basin^30^. The circulation in this region is also characterized by an extremely high variability due to the confluence of two surface flows: a portion of the Liguro-Provençal-Catalan Current (Northern Current) that flows southward, and a northward branch of the Atlantic inflow circulating along the eastern side of the channel, producing an intense frontogenetic activity^30–32^. The phytoplankton production in this area is not limited by sunlight but by nutrients availability, following a pattern of phytoplankton variability typical of oligotrophic regions, with maxima in winter and minima in summer and intermediate values during spring and autumn^33,34^.

The natural approach to study the transport of tracers in the ocean is the Lagrangian perspective. A wide range of Lagrangian techniques has emerged in the last two decades, most of them focused on the extraction of the Lagrangian Coherent Structures (LCS) of the flow to provide a fluid transport radiography (Haller *et al*.^35^, and references therein). Here we use the Finite-Size Lyapunov Exponents (FSLEs)^36–39^ to reveal surface LCSs as well as to provide the relevant spatio-temporal scales of dispersal, thus giving information on the horizontal mixing activity. At coastal scales, the dynamical picture in the Lagrangian frame has been studied using velocity data from models^40–42^ as well as from HFR^43–46^. Transport properties of the flow can also be characterized analyzing the evolution of surface areas, by computing either the divergence along the fluid particle trajectories^47^ or the evolution of numerical particle triads^48^ or drifters triplets^24^. Recent studies using velocities from models have shown the relation of surface clustering with areas of high values of Eulerian^19^ and Lagrangian^49,50^ divergence.

We investigate here the small scale transport processes (3.5–25 km) based on direct observations of high resolution surface currents and their relationships with phytoplankton concentrations in a coastal region. We introduce a recently developed metric called Lagrangian divergence adapted for finite domains that, together with the well-established LCSs, are used to explain the distribution of surface chlorophyll. We show that those complementary Lagrangian diagnostics computed from HFR currents are able to infer coastal transport processes and their influences on biological activity with unprecedented details.

## Results

### Relevance of small scale processes to properly map coastal transport

We first provide evidences to support the reliability of the HFR currents for our computations. The Lagrangian validation has been performed using data from 8 drifters trajectories available in the domain of interest (HFR area of coverage) during the period of study (July 2012–July 2014). We compare synthetic drifter trajectories integrated in the HFR velocity field and real drifter trajectories by computing the distance (D) between each virtual drifter trajectory and the real one^51^. The largest values of D were found for drifters deployed in October 2012 and April 2013 reaching a separation distance of 7–8 km after 24 hours (see Fig. S2 a in Supplementary Information). The smallest D values (~1 km) were found for the drifters deployed in September 2012. This difference in the D values could show the different dynamical conditions between both months. Nevertheless, the mean distance of separation, i.e. averaging over all the trajectories, after 24 hours is about 4 km (see Fig. S2b). It demonstrates a good accuracy of the HF Radar measurements as well as of the particle trajectories derived from it.

**Figure 1:**
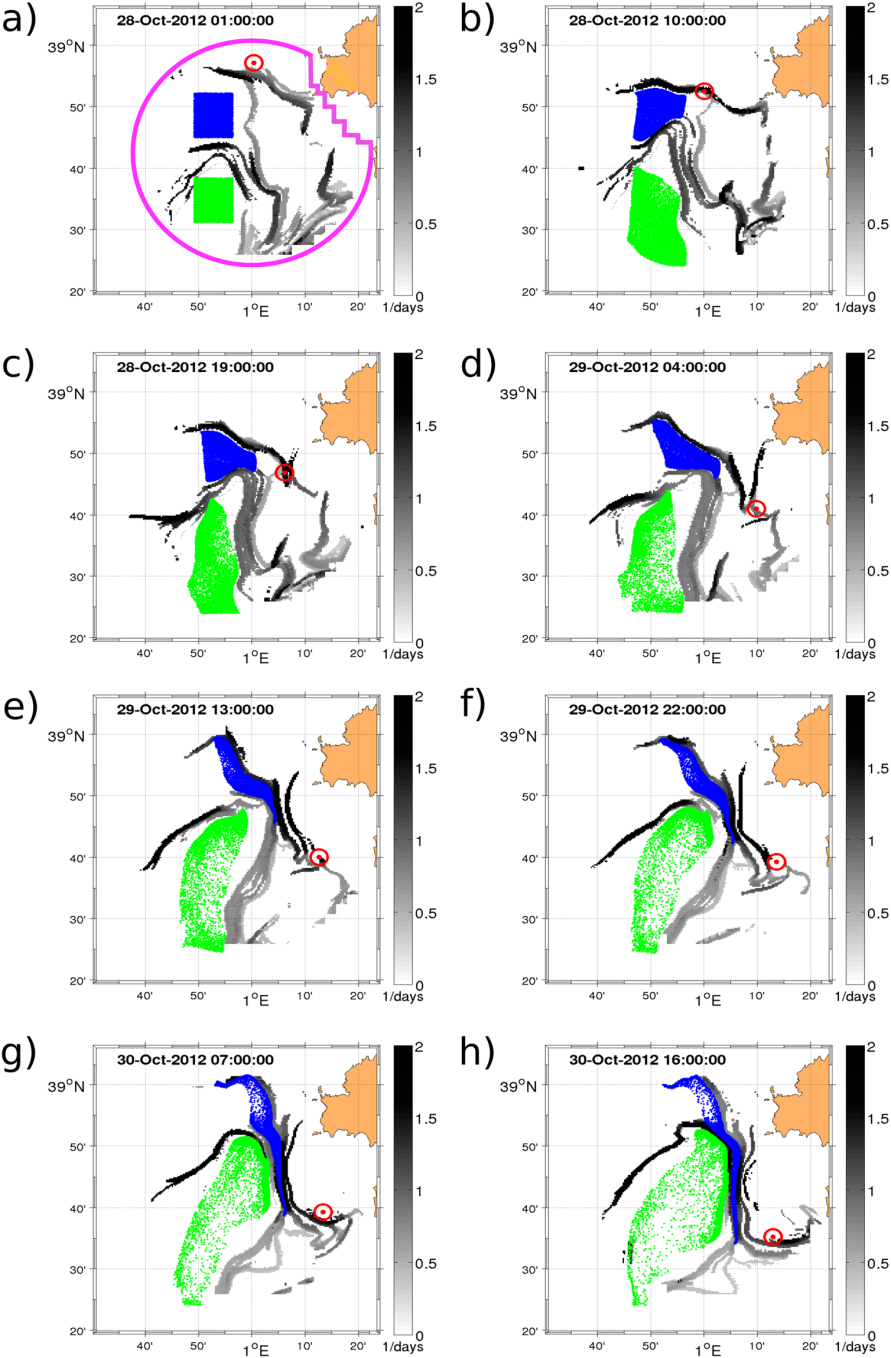
Evolution of two sets of particles (green and blue) and real drifter (red dot-circle) in the area covered by the HFR (magenta annotations in panel a) in October 2012 superimposed on the backward FSLE (grey colorbar). The virtual particles are initially deployed at both sides of a barrier revealed by a zonal LCS on October, 28th, 2013 at 00:00. The figure was made using MATLAB R2012a (http://www.mathworks.com).

The performance of HFR-LCS (Lyapunov ridges extracted from HFR velocities, -see Materials and Methods) in structuring the flow is shown by advecting in the HFR velocity field two sets of virtual neutrally buoyant particles initially deployed on the northern and southern flank of a given LCS that lays across the area from east to west in October 28, 00:00 UTC (Fig. 1a). Although the location and magnitude of this LCS evolve in time, the LCS persists for several hours manifesting the presence of a coherent transport barrier preventing both sets of particles to be mixed up. A meridional LCS is formed and maintained during the simulated period, limiting water exchanges between the coast and the open ocean (Fig. 1a–h). The significance of these HFR-LCS in organizing the transport in this coastal region is also supported by comparing their evolutions with real drifters trajectories. It constitutes an independent and observed dataset to further validate our approach. In Fig. 1, the position of a real drifter is superimposed on the backward FSLE field at every snapshot. The drifter is initially located close to an attractive LCS and it remains in the vicinity of the structure during the entire period. The same validation has been performed for other 7 drifters with similar results (see Movies in Supporting Information). To evaluate how backward LCSs constrain the drifters motions we have compared the probability distribution function (PDF) of the values of the backward HFR-FSLEs at the position of the real drifter with the PDF of the FSLE values for the entire HFR domain (Fig. S1c). As compared with the peak at low FSLEs found in the rest of the domain, a clear peak of the PDF at high FSLE is found at the drifters locations. This larger occurrence of high FSLE at the drifter positions shows that drifters are attracted to backward LCSs. Note that in contrast to other authors^51,52^ we use Lagrangian averaged metrics, i.e. LCSs, instead of trajectories to validate the HFR velocity field and the derived Lagrangian computations.

Both tests based on virtual particles and using real drifters demonstrate that even for restricted domains (~thousands of km^2^) and small temporal scales (few hours to a few days), the LCS diagnostics obtained from the FSLE computation applied to high-resolution HFR surface currents are robust representations of surface dynamics in coastal basins.

**Figure 2:**
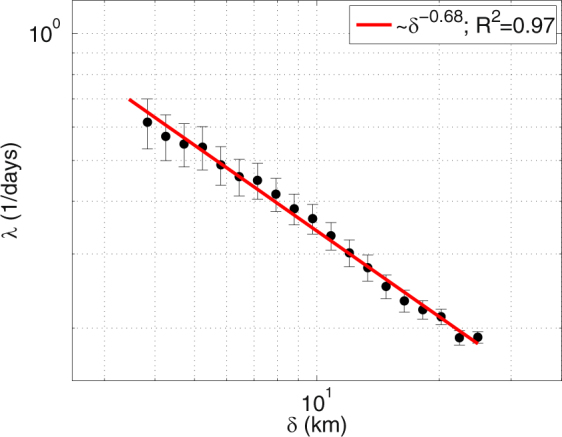
FSLE curve (*λ*(*δ*)) at different spatial scales $\delta$ using velocity data from HF Radar averaged over a total of 480 samples homogeneously distributed from July 2012 to June 2014. The legend shows the value of the slope and the correlation coefficient obtained from the best fitting analysis. The figure was made using MATLAB R2012a (http://www.mathworks.com).

Following other authors^17,45,53^ we now evaluate the dynamical importance of the scales captured by the HFR by computing particle dispersion at different spatial scales using Eq. 1 (see Material and Methods section). The averaged FSLE curve (Fig. 2) shows that *λ*(*δ*) is not constant over the range of scales covered by the HFR (scale dependent). The best-fitting of the non-averaged (or individual) FSLE curves returned slopes spanning −0.64 to −0.73; the best fit of the averaged FSLE curve shows a slope of −0.68 with a correlation coefficient, R^2^, of 0.97. This indicates that the average scaling law of the relative dispersion over the scales ranging from 3.5 km to 25 km is associated to a Richardson turbulent diffusion^54,55^. It means that the main contributors to the separation rate are structures with size comparable with the separation itself, therefore the dispersion regime is local.

This shows that the lateral relative dispersion, at the surface, is governed locally by small-scale structures and not only by meso- and larger-scale structures. It also suggests that HFR is partially resolving submesoscale i.e. it is capturing the largest (~3.5–10 km) submesoscale features of the flow. Targeted fitting analysis on specific scales shows that over 3.5–8 km the slope is smaller (−0.60) than for scales ranging 8–25 km (−0.71). The dependence of *λ*(*δ*) at *δ* larger than 25 km cannot be explored due to the limited area covered by the HFR. Note that those scales (<25 km) are not resolved by the geostrophic velocities inferred from altimetric Sea Surface Height (SSH), thus the use of HFR velocity fields is necessary to accurately study transport processes in this region. From the fitting in the Richardson regime (*λ*(*δ*) ~${\varepsilon }^{\mathrm{1/3}}\cdot {\delta }^{-\mathrm{2/3}}$)^55^ we obtain a mean turbulent dissipation rate *ε* of 2.5 $\cdot {10}^{-9}{{\rm{m}}}^{2}/{{\rm{s}}}^{3}$, which is similar to values reported in most oceanic regions by Corrado *et al*.^17^, using drifters.

The slope of the FSLE curve for synthetic drifter trajectories integrated with HFR velocity field is consistent with previous observational and modeling studies, with a slope similar to those derived from surface drifters^16,26,56^ and from numerical simulations^22^. It further reinforces the fact that the velocity field of the HFR is adequate to study dynamics over the upper submesoscale (3–10 km) and the lower mesoscale (10–30 km).

The *λ* obtained in our computations is one order of magnitude smaller than the one of Haza *et al*.^45^, (*λ* = 4 to 7 ${{\rm{d}}{\rm{a}}{\rm{y}}{\rm{s}}}^{-1}$ for *δ* < 1 km) computed from VHF Radar with 300 m of spatial resolution in the Gulf of La Spezia. This is expected when comparing their scales of interest [0.1–1 km] against our range of resolved scales [3.5–25 km]^17^. Comparing with other observational studies in the Mediterranean Sea and at this range of scales we found values of the FSLE curves of the same order (O(1) days^−1^). Schroeder *et al*.^56^, using drifters deployed in the Ligurian Sea reported slightly larger values of *λ* (between 0.9 and 0.4 days^−1^) at spatial scales between 3 and 30 km following a Richardson slope. In the Adriatic Sea similar values of *λ* (0.7–0.2 days^−1^) were reported for the scales range studied here^26^. Other studies based on drifters in the Gulf Stream^15^ have found higher values of *λ*(*δ*). In addition of possible observational biases, various regional discrepancies could explain these different values of FSLE. To name just a few, a strong coastal jet, alternating tidal currents, prominent topographic boundaries, specific wind regimes, etc… will influence transport dynamics and/or affect near-surface measurements.

### Impact of lateral stirring on coastal Chl-a distribution

**Figure 3:**
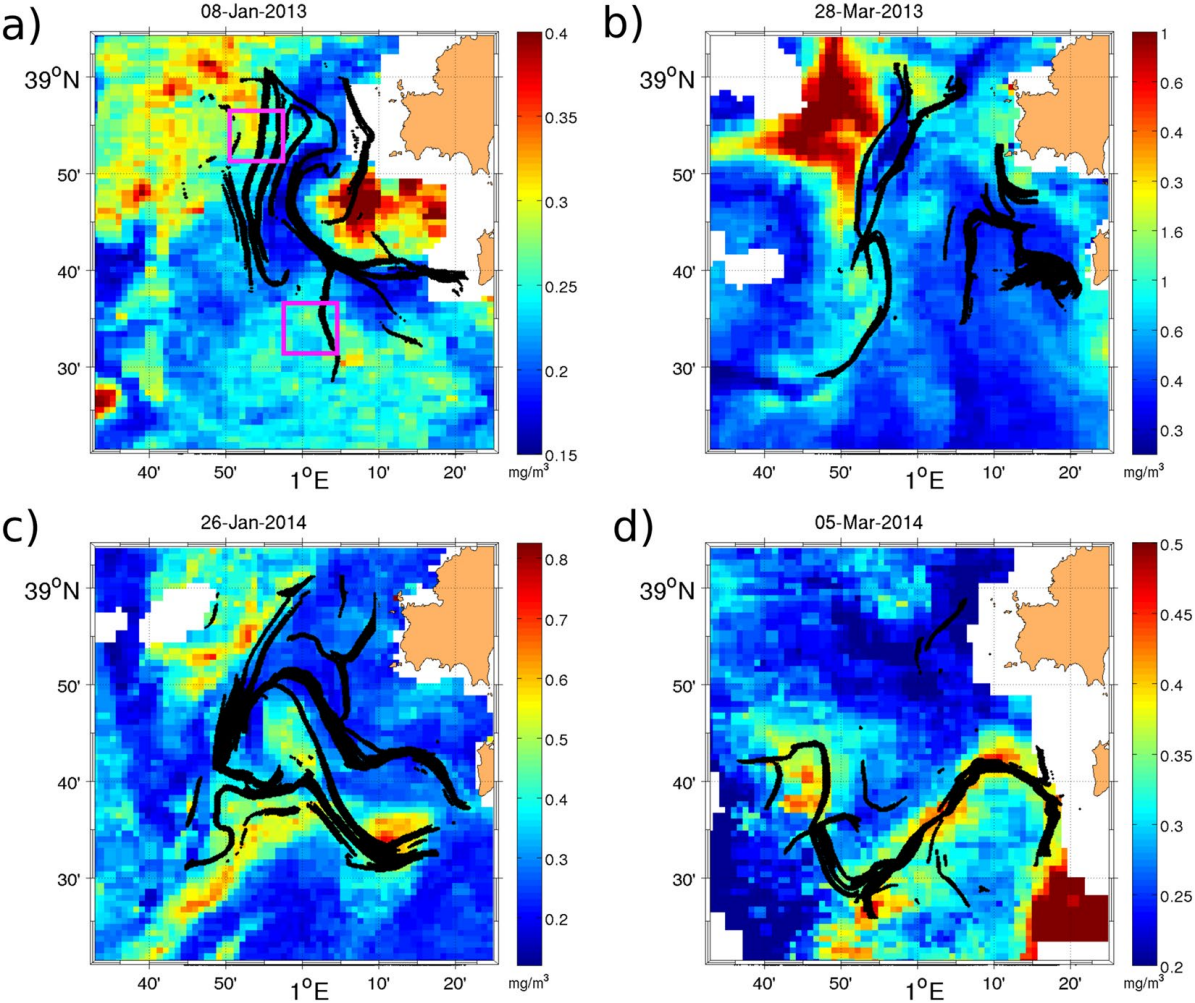
LCS superimposed to CHL-a maps for (**a**) January 8th 2013, (**b**) March, 28th 2013, (**c**) January 26th, 2014 and (**d**) March 5th, 2014. Magenta rectangles represent the northern and southern sub-domains ($8\times 8$ km). The figure was made using MATLAB R2012a (http://www.mathworks.com).

Next we characterize the biological response to transport processes by comparing the HFR-LCSs with patterns of satellite Chlorophyll-a (Chl-a) at 1 km resolution as a proxy of surface phytoplankton concentrations. Figure 3 shows four snapshots of the spatial distribution of surface Chl-a together with the HFR-LCSs (in black) corresponding to winter and early spring of 2013 (Fig. 3a and b) and 2014 (Fig. 3c and d). Spatial distribution of Chl-a is very heterogeneous within this relatively small area, exhibiting important phytoplankton patchiness. As observed in each snapshot the attractive LCSs shape the spatial distribution of Chl-a. High concentrations of Chl-a are constrained (Fig. 3a and b) and stirred (Fig. 3c and d) by Lyapunov lines.

**Figure 4:**
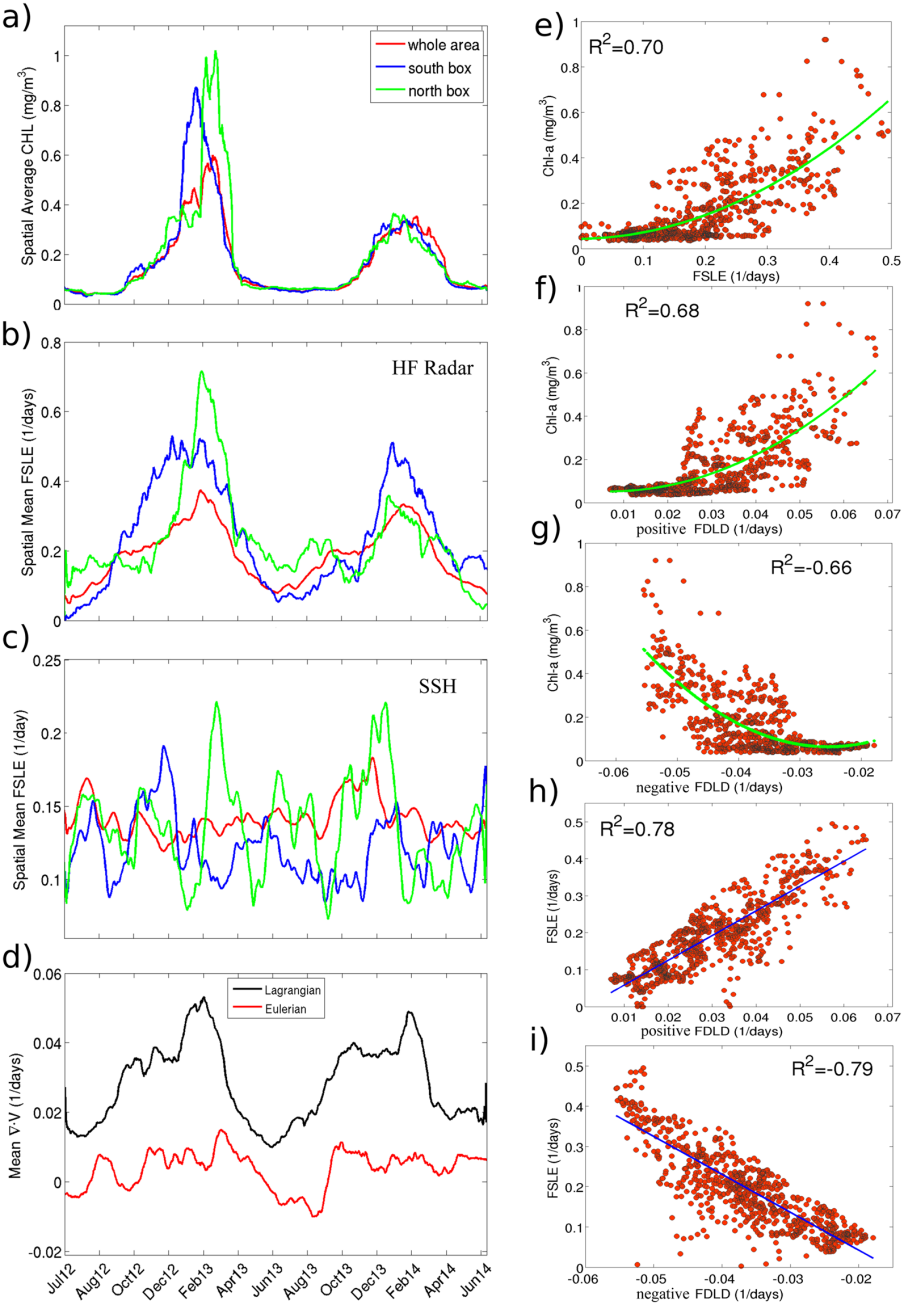
Time-series from July 2012 to June 2014 of daily means of (**a**) Chl-a concentration (in mg/m^3^), (**b**) backward HFR-FSLE, (**c**) backward SSH-FSLE. The red line corresponds to the spatial mean for the area covered by the HFR and the blue and green lines for two control boxes shown in Fig. 3a by the magenta squares. (**d**) Time-series from July 2012 to June 2014 of daily means of Eulerian (red line) and Lagrangian (black line) divergence. Scatterplots between daily spatial averages of (**e**) Chl-a *vs*. backward HFR-FSLE, (**f**) Chl-a *vs*. positive FDLD (**g**) Chl-a *vs*. negative FDLD (**h**) forward HFR-FSLE *vs*. positive FDLD (**i**) backward HFR-FSLE *vs*. Negative FDLD.

Phytoplankton blooms are commonly observed in this region during winter and early spring^33,57^. Figure 4a shows the temporal evolution of the daily Chl-a concentration spatially averaged over the whole HFR area (magenta annotation, Fig. 1a). We also display the time-series averaged over small northwestern and southeastern boxes (magenta boxes, Fig. 3a) to study the spatial propagation of the bloom. Boxes are chosen following the main circulation pattern (northwestward) given by the first mode of the EOF analysis of the HFR currents^29^. As observed in the southern control box, the maximum of Chl-a appears at the end of January 2013 and for the northern control box at the end of February - early March. A less intense bloom is also present during 2014 with a slight shift of the maximum between the two control areas. A possible scenario is that nutrients-enriched coastal waters are advected by the northward flow of Atlantic waters, intruding the HFR area and mixing (small-scale structures) with the nutrient depleted Liguro-Provencal-Catalan current.

As a measure of mixing activity, we compute spatial means of FSLE values for the whole region and for both control areas (Fig. 4b). FSLE maxima coincide with maxima of Chl-a. Large FSLE values appear first in the south box and then in the northern one, perfectly matching the meridional order of appearance of Chl-a maxima. The best non-linear fitting between daily Chl-a and FSLE values over 2 years gives a correlation coefficient of 0.70 (Fig. 4e) for a polynomial fitting of second order. High mixing activity produced by the stirring of surface currents is involved in the generation of water masses with large values of phytoplankton. This has been also observed by Calil and Richards^11^, where large distribution of phytoplankton in oligotrophic areas was related to the dynamical mesoscale structures inferred from altimetry. By contrast, other studies showed that large surface mixing activity has a negative effect on phytoplankton productivity in upwelling regions^12,42^, which was explained as a “regional-scale” lateral-induced loss of nutrients^14^. This strong positive correlation between FSLE and Chl-a is an indicator of the tight bio-physical interactions at the small-scales revealed by HFR. Other factors could be involved in the biomass enhancements, namely, the seasonal development of the mixed layer depth (MLD) and of the thermocline. Lavigne *et al*.^34^, based on observations have reported that this region exhibits a very shallow MLD in summer, reducing the supply of nutrients from deep waters, and a deeper MLD in winter, as a consequence of the specific atmospheric conditions, favoring the fertilization of surface layers. Further comparison of the MLD with the nutricline and euphotic depth has shown that primary production was limited by nutrient fueling from below rather than by light availability^34^. This suggests that the growth of phytoplankton is controlled by either a MLD deeper than the nutricline, or by the vertical advective transport of nutrients induced by the small scale processes within the mixed layer. Indeed, previous works have shown that the supply of nutrients to the euphotic layer is related to the submeososcale processes, i.e., the stretching of filaments through horizontal advection (see Mahadevan^7^ for a review). While our results clearly suggest that, in this coastal region, high mixing activity is associated with abundant phytoplankton, the precise mechanisms remain unknown.

We also computed the FSLE using geostrophic currents from Altimetry (SSH-FSLE) (see Materials and Methods) and we found that the temporal evolution of spatially averaged SSH-FSLE did not capture the seasonal change of mixing activity seen by the HFR (Fig. 4c). No significant correlation is found between the averaged values of geostrophic FSLE and Chl-a over the whole region nor the averaged values of both control areas.

### Accumulated effect of small scale sea surface distortion on phytoplankton patchiness

To further study the mechanisms explaining the relationship between phytoplankton patchiness and transport processes, we now exploit a geometrical quantity related to the contraction and expansion of the flow. If a three-dimensional flow is incompressible then, following the continuity equation, the three-dimensional divergence of the velocity field is zero and one can use the two-dimensional horizontal divergence at the surface to differentiate areas of contraction and expansion of the fluid flow. If the ocean flow is strictly two-dimensional at the sea surface, divergence will be zero and the corresponding ridges of the FSLE field correspond to lines of stretching and folding. However, in truly 3D flows, when vertical motions exist, the horizontal divergence will be non-zero and the horizontal convergence/divergence zones at the surface are related to stretching and folding areas as well as to vertical motions. Positive divergence indicates upward motion and negative divergence is associated with downward motion. These conclusions do not hold for altimetry-derived velocities because the geostrophic approximation results in horizontal currents (neglected vertical velocities) of zero-divergence.

**Figure 5:**
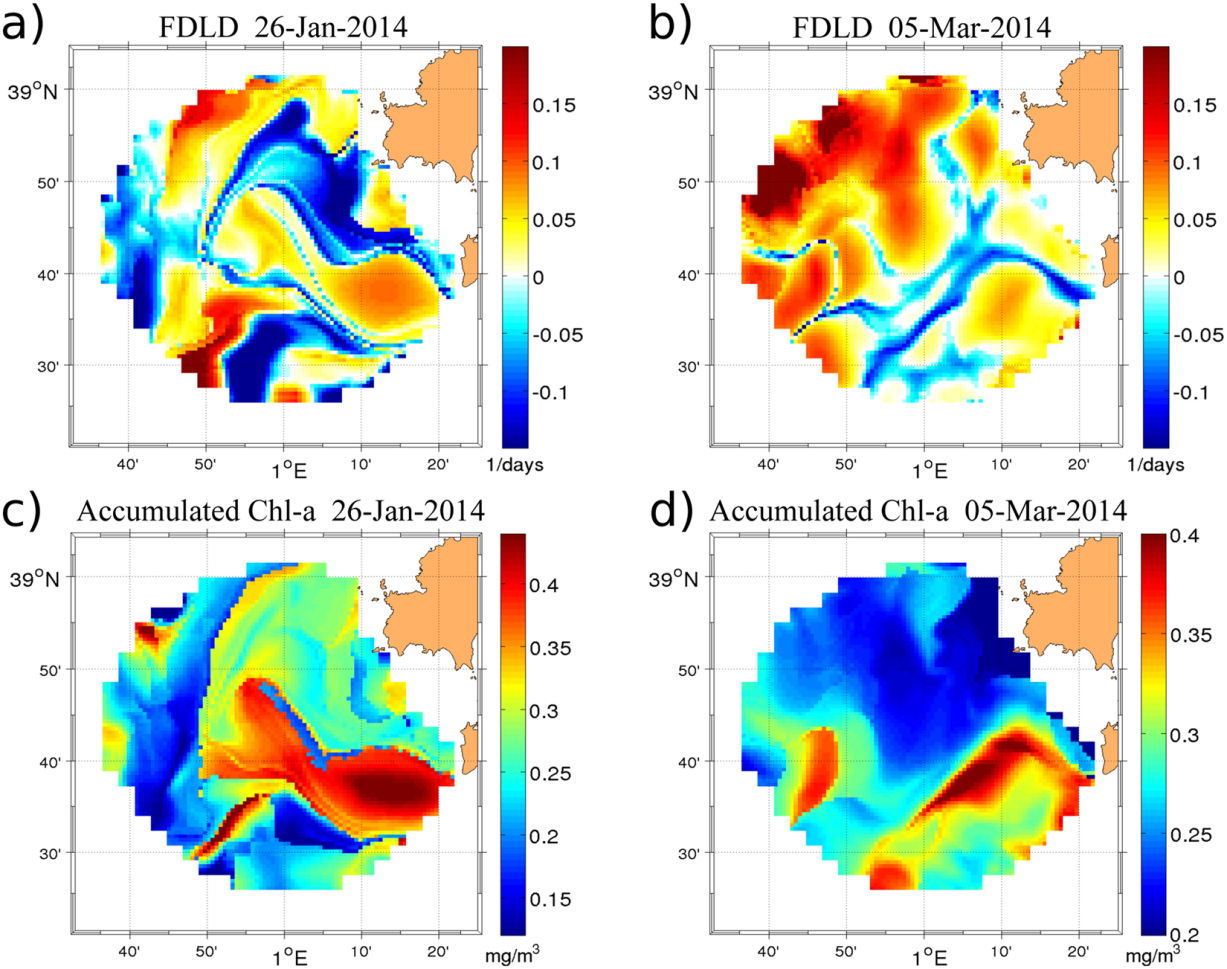
Top panel: Snapshots of FDLD (in days^−1^) from HF Radar corresponding to (**a**) January 26, 2014, (**b**) March 5, 2014. Bottom panel: Accumulated Chl-a (in mg/m^3^) computed for the same time instant that in top panels. (**c**) January 26, 2014, (**d**) March 5, 2014. The figure was made using MATLAB R2012a (http://www.mathworks.com).

Assuming that the divergence of surface velocity field derived from HFR data is non-zero, we analyze the accumulated effect of convergence and divergence of a fluid parcel by computing the Finite-Domain Lagrangian Divergence (FDLD) given by Eq. 5 (see Materials and Methods). The integration duration is chosen according to the typical time-scales required for phytoplankton communities to respond to a new supply of nutrients, which is around 5 days in frontal systems^58^. Snapshots of FDLD for two different days displayed in Fig. 5(a and b) show complex patterns, attesting of the high spatial variability of convergence and divergence zones.

Comparing spatial patterns in Figs 3d and 5b, we find that convergence lines given by minimum (negative) values of FDLD (ravines in FDLD field) identify attractive LCSs, thus revealing dynamical structures that affect transport. Figure S3 in Supporting Information shows further exemplary comparisons between both Lagrangian structures. Figure 4i reveals a linear fit as the best fit found between both dynamical (backward FSLE) and geometrical (FDLD) spatially-averaged quantities with a negative correlation (*R*^2^ = −0.79). Negative values of FDLD suppress expansion and enhance trapping, giving rise to regions of clustering. In those regions, high values of Chl-a are concentrated along the negative lines of FDLD (Figs 3d and 5b) which match the attractive LCS. In this case also a polynomial fit of second order was found as the best fit between Chl-a and negative FDLD with a correlation of *R*^2^ = 0.66 (Fig. 4g). Note that since FSLE ridges also identify regions of high shear, only the well-matched patterns of FDLD and FSLE can be used to distinguish local contraction and expansion motions. The numerical study by Jacobs *et al*.^50^, from models at different resolutions reveals that surface material clustering is initially dominated by these small scale features. We have seen here how the clustering effect of small scale processes contained in HFR currents can affect horizontal phytoplankton distribution.

Next we explore the effect of vertical motions associated to the dynamical structures derived from LCS and FDLD on phytoplankton distribution. Calil and Richards^11^ showed that regions of pronounced gradients of relative vorticity, related to backward FSLEs, are expected to exhibit large vertical velocities. The scatterplot displayed in Fig. 4h shows good correlations between spatial averaged forward FSLE and positive FDLD (*R*^2^ = 0.78). The effect of these geometrically-induced vertical motions on phytoplankton concentrations is shown in Fig. 5a. We find that regions of large Chl-a concentrations (Fig. 3c) are associated to regions of positive FDLD values (Fig. 5a) that indicate accumulated upward vertical velocities. Two distinct mechanisms could explain the fact that extreme FDLD values (both positive and negative) are associated with phytoplankton patches. Positive FDLD extrema indicate divergence and the predominance of vertical upward motions of subsurface nutrients, thus promoting locally phytoplankton growth. Negative FDLD extrema mean convergence and horizontal clustering, thus accumulating surrounding phytoplankton. To disentangle vertical and horizontal dynamics and to determine the preponderant mechanism, our results advocate for carefully analyzing the sign of the FDLD extrema as well as the relationship between FSLE and FDLD fields.

Time-series of the spatial averages of FDLD (Fig. 4d) show that maxima of FDLD associated to high positive divergence (upward motions) occur concomitantly with the growth of phytoplankton (Fig. 4a) and with the peak of mixing activity (Fig. 4b). This notable seasonal variability of FDLD is not seen for Eulerian (instantaneous) divergence (Fig. 4d). Positive Lagrangian divergence prevails over negative values during the phytoplankton bloom periods. The scatterplot of Chl-a *vs*. FDLD displayed in Fig. 4f shows the best non-linear fitting (polynomial fit of second order) with a positive correlation between both quantities (*R*^2^ = 0.68).

Note however that this significant correlation between Chl-a and FDLD does not hold when the comparison is made pixel-by-pixel. This misfit could be due to the fact that those observations (currents and Chl-a) are not exactly concomitant as they originate from different remote sensors or to the tentative comparison between an Eulerian (Chl-a) and a Lagrangian (FDLD) quantity. Also daily Chl-a concentrations derived from satellite satellite suffer from gaps due to cloudiness (thus reducing the number of pixels available for the comparison). To circumvent this issue, we adopted a novel methodology inspired from the Lagrangian framework: we integrated the Chl-a scalar field along the trajectories provided by the HFR during the same integration period of the FDLD to estimate the “cumulative amount of positive FDLD” seen by each patch of Chl-a along its drift. The correspondence between both variables is improved when comparing two snapshots of accumulated Chl-a (Fig. 5c and d) with the corresponding FDLD patterns (Fig. 5a and b, respectively). Regions where the accumulated Chl-a is large match well regions of high negative FDLD values (where clustering is strong) as well as high positive FDLD values (where upward motions are predominant). To quantify the relationship between vertical motions and phytoplankton concentrations we compute the correlation coefficient between values of accumulated Chl-a and values of positive FDLD. We obtain a value of *R*^2^ = 0.56, which is much larger than when using the Eulerian Chl-a (*R*^2^ = 0.25).

## General Discussion and Conclusion

We investigate coastal transport using two complementary Lagrangian diagnostics, FSLE and FDLD, both computed on a high-resolution velocity field derived from HFR. The combination of both quantities allows us to distinguish regions of high shear, convergent regions where clustering dominates, and areas with persistent vertical motions. These analyses are then used to characterize the physical processes which impact biological activity and patchiness in coastal regions.

The scale-dependence of HFR-FSLE provides an evaluation of the size of the dynamical features captured by the HFR affecting the transport properties. The slope of the averaged FSLE curve over the scales comprised between 3.5 km and 25 km is associated to a Richardson-like dispersion. This slope is in agreement with previous observational studies with drifters^16,26,56^. At the range of scales measured by HFR, surface relative dispersion is locally controlled by structures in the upper submesoscale range (~3.5–10 km) and in the lower mesoscale (10km–25km).

HFR-LCSs are prominent structures that organize transport and dispersion of coastal waters in the Ibiza channel, impacting physical and biological coupled processes at regional scales. HFR-LCSs constrain the motion of virtual particles and real drifters, and shape the distribution of Chl-a concentration. Lehahn *et al*.^9^, showed that mesoscale fronts obtained from SSH-FSLE separate regions of different Chl-a concentrations in the open ocean. However, in coastal basins, SSH-FSLE is not able to capture the seasonality of the mixing activity nor the spatial distribution of LCSs provided by the HFR-FSLEs. This reveals that small scale processes are important to understand phytoplankton patchiness in coastal basins. The influence of small-scale coastal dynamics on surface phytoplankton distribution in coastal areas can be properly studied considering (i) small spatial-scales (${0.0345}^{\circ }$ *vs*. ${0.125}^{\circ }$ resolutions of HFR and SSH, respectively), (ii) high frequency data (hourly *vs*. daily velocities of HFR and SSH, respectively) and (iii) the total velocities (geostrophic + wind-driven + Stokes drift) currents of HFR data *vs*. geostrophic component of the SSH velocities). As an additional example, we compare the SSH-LCSs with the HFR-LCSs (see Fig. S4a and S4b in SI) at the same spatial resolution of 0.4 km. Although both computations identify an eddy-like structure, the one derived from altimetry is shifted westward as compared to the eddy detected by HFR. Similarly, the finer filamentary structure provided by HFR-FSLE is not well described by SSH-FSLE.

Using FDLD, we show that such small scale structures can be further distinguished in those that likely produce clustering and those that are rather associated with large accumulated upward velocities. On one hand, regions of highly negative values of FDLD are convergent and prone to clustering; thus they accumulate the surrounding phytoplankton standing stocks. On the other hand, regions of positive FDLD are associated to the prevalence of upward vertical velocities that promote new production of phytoplankton due to enhanced nutrient supply. By comparing the fields of our dynamical Lagrangian diagnostics with high resolution maps of Chl-a, we found that regions of high Chl-a match regions with both positive and negative extrema of Lagrangian divergence, while only the latter coincide also with attractive LCSs. In our area of study and over typical time-scales of a few days, positive divergence predominates during the periods of phytoplankton blooms in fronts, suggesting that accumulated upward velocities produced by the highly divergent horizontal flow favor phytoplankton growth through the upwelling of subsurface nutrients, thus controlling the biological dynamics.

The relationship between the physical (FSLE and FDLD) and the biological (Chl-a) variables is first evaluated through the significant correlation coefficients found by comparing spatially averaged values. The best fitting analysis between both variables shows good correlations for polynomial fitting of second order. This suggests that stirring (FSLE) and flow divergence (FDLD) have significant effects on phytoplankton distribution when they reach extreme values; i.e. when mixing activity and divergence/convergence are high, only high values of Chl-a are observed. When the mixing activity or the divergence/convergence are low to moderate, their effects on phytoplankton is not so clear. The relationship between these two physical drivers and Chl-a is therefore symmetric; they can be viewed as enhancing factors for planktonic biomasses.

Furthermore we explored the spatial agreement of the FDLD with Chl-a estimating the Lagrangian accumulation of Chl-a along the trajectories of the fluid parcels. We go to the accumulation of Chl-a because we want to see the accumulative process of nutrients injection. This approach returned a much better correlation between both variables than using the “Eulerian” Chl-a. Of course a tight absolute relationship between Chl-a and FDLD is not expected since many other key biological processes for phytoplankton (e.g. heterogeneous light field, recycled production, zooplankton grazing, viral infections, etc…) were not taken into account here.

As ocean surface currents forecasting systems are being developed^5,59^, our methodology and the diagnostics exploited here could be applied on current predictions and thus serve to project future spatial distribution and magnitude of Chl-a in the coastal ocean. More generally, our study proves that the combination of both LCSs and FDLD computed from HFR is a powerful framework to study the effect of transport on biological quantities in coastal seas, as well as to localize convergence/divergence zones which are relevant for the tracking of debris accumulation or jellyfish aggregations, and for other coastal management activities, such as search and rescue missions and oil spill management.

## Materials and Methods

### HF Radar measurements and drifter data

A coastal HFR network combining two CODAR antennas in the Ibiza Channel (IC) area provides hourly velocities up to 70 km from the coast of Ibiza with a radial resolution of 1.6 km (data available at http://www.socib.es). The coverage area of the HFR is shown by the magenta circle plotted in Fig. 1a. Cartesian velocities are provided for the period of June, 1st 2012 to July, 31st 2014 in a regular grid of 393 points over an area of approximately $3500\,{{\rm{km}}}^{2}$ with a resolution of 3 × 3 km and with an accuracy of ±0.5 m/s^29^. In order to be able to compute particle trajectories the gaps of the HF Radar velocity field have been filled using the OMA (Open-boundary Modal Analysis) algorithm developed by Kaplan and Lekien^60^.

Drifter data used for the validation of the HF-Radar velocities and HFR-LCS are part of different experiments performed by ICTS SOCIB (http://www.socib.es) in the Ibiza Chanel. Drifters position were provided through GPS positioning transmitted via GSM every 5 minutes.

### Satellite data

We use daily AVISO Sea Level Anomalies at 1/8° of spatial resolution derived from a Ssalto/Duacs multimission altimeter regional product released in 2016 to compute the Absolute geostrophic surface velocities. This product can be downloaded from (http://www.aviso.altimetry.fr/en/data/products/sea-surface-height-products.html). It consists of surface currents computed using time-variable Mediterranean Absolute Dynamic Topography ($\eta$) calculated from mapped altimetric sea level anomalies combined with a mean dynamic topography computed for the Mediterranean Sea^61^. Then the zonal and meridional components of the Absolute geostrophic currents, (${{\rm{U}}}_{\varphi },{{\rm{V}}}_{\theta }$), are obtained from the geostrophic relationship. The velocity field used here covers a period from June 2012 to June 2014.

We use ocean surface Chlorophyll data obtained from the ESA-CCI Remote Sensing Reflectance spectrum using a specific regional processing algorithm for the Mediterranean (MedOC4). The reflectance is the result of merging MODIS-Aqua, SeaWiFS and MERIS sensors. The product is distributed by CMEMS and can be downloaded from http://marine.copernicus.eu. This product measures the average chlorophyll content over the optical depth. Gridded daily data were used with a spatial resolution of approximately 1 × 1 km.

### Finite Size Lyapunov Exponents and Coastal Lagrangian Coherent Structures

We quantify horizontal transport processes by the Lagrangian technique of the Finite Size Lyapunov Exponents (FSLEs)^36,62^. FSLE is specially suited to characterize flows with multiple spatio-temporal scales^53,63^, and to study the stretching and contraction properties of transport in geophysical data^38,64^.

FSLE was originally introduced in the dynamical system theory to characterize the growth of non-infinitesimal perturbations in turbulence^36^ by the following equation,

1 $$\lambda (\delta )=\frac{\mathrm{ln}(r)}{\langle \tau (\delta ,r\delta )\rangle },$$

where $\langle \tau (\delta ,r\delta )\rangle$ is the time (*τ*) needed for the initial perturbation *δ* (pair-particles separation) to grow r *δ* averaged over all the particles pairs for every initial perturbation *δ* and a fixed threshold rate, *r*.

The explicit dependence of this expression of average FSLE on the separation scale *δ* allows to isolate the contribution of different spatial scales to particle-pair separation^53,63^, providing a characterization of the relative dispersion processes in terms of scaling laws. In contrast to relative dispersion metrics treating the time as the independent variable, *λ*(*δ*) (spatial scale, r*δ*, as independent variable) is not affected by the interference of pair-particles at different dispersion regimes. The value of *r* has to be small (*r* ¡ 2), in order to capture the relative dispersion from small coherent features and avoid the contribution of different scales of motion in the average, and not close to 1 to avoid aliasing problems related to the time step of particle advection at small scales^63,65^.

The scaling laws associated with the different relative dispersion regimes are:^17,53,63,66^ (i) $\lambda (\delta )\sim$ constant, for exponential separation between particles associated with non-local chaotic advection^53^; (ii) $\lambda (\delta )\sim {\varepsilon }^{\mathrm{1/3}}{\delta }^{-\mathrm{2/3}}$, for ocean turbulence cascade leading to Richardson scaling, where ${\varepsilon }^{\mathrm{1/3}}$ is the mean turbulent dissipation rate^55^; (iii) $\lambda (\delta )\sim {\delta }^{-1}$, for ballistic or shear dispersion produced by constant velocities differences associated with particles moving along separated currents; and (iv) $\lambda (\delta )\sim {\delta }^{-2}$, for diffusive scaling associated to uncorrelated pair velocities.

We compute the averaged FSLE with a total of 648 particle pairs trajectories released in the area of HF Radar velocity field for each *δ* ranging from 3.5 km to 25 km, and r = $\sqrt{2}$. We repeat the same computations for 480 different initializations (homogeneously distributed through the two years of data). Particles escaping the HF Radar domain are not taken into account in the average computation. Only original pairs have been taken into account for the average. The particle trajectories in a 2*D* HFR velocity field are governed by,

2 $$\frac{d{\bf{r}}}{dt}={\bf{v}}(x,y,t),$$

where ${\bf{v}}=(u,v)$ are the zonal and meridional velocities measured at coordinates ${\bf{r}}=(x,y)$ by the HFR.

FSLE can also be used to unveil dynamical structures that act as transport barriers^37–39^. The calculation of the LCSs from FSLEs goes through computing the minimum time, $\tau$, required for two fluid particles initially centered in **r** and separated by a distance *δ*_0_ to reach a fixed final separation distance *δ*_*f*_. At position **r** and time *t*, the FSLE, denoted by *λ*, is given by:

3 $$\lambda ({\bf{r}},t,{\delta }_{0},{\delta }_{f})=|\tau {|}^{-1}ln\frac{{\delta }_{f}}{{\delta }_{0}}.$$

In this definition of FSLE ${\delta }_{0}=\delta$ and ${\delta }_{f}=r\delta$ using a large value of *r* to adequately distinguish regions of extrema of the FSLE field. Note that, to have an explicit space-time dependence, averages are not performed here, in contrast with the original definition 1. The largest Lyapunov values concentrate along characteristic lines, Lyapunov lines, which can approximate manifolds of relevant hyperbolic points, the so-called Lagrangian Coherent Structures (LCS)^35,67^. Fronts, eddies and filamentary barriers to transport can be identified with these manifolds. Since LCS cannot be crossed by particle trajectories, such lines strongly constrain and determine fluid motion, organizing ocean transport.

To estimate the LCS from FSLE computations we use the algorithm described in Hernandez-Carrasco *et al*.^39^, adapted to finite domains, in this case the area of HFR coverage. The minimum time *τ* is computed by integrating the trajectories of the four neighboring points of the analyzed one located at **r** and by selecting the associated particle that separates faster *δ*_*f*_. The grid points whose four neighboring trajectories escape the domain before separating *δ*_*f*_ are not taken in the analysis. The relation between *δ*_0_ and *δ*_*f*_ has been calibrated through previous experiments in order to obtain clear and robust LCSs in the relatively small domain of the HFR coverage. The total time of integration is chosen to be greater than the maximum value of the average residence time obtained in this area. In our computations the residence time is always less than 15 days so we used a maximum of 15 days for integrating the particle trajectories. We compute the FSLEs for all points **x** in a lattice of initial conditions, defined by a grid spacing coincident with *δ*_0_.

Numerically, we integrate Eq. (2) using a standard, fourth-order Runge-Kutta scheme, with an integration time step $dt=10$ minutes. Since information is only provided in a discrete space-time grid, spatio-temporal interpolation of the velocity data is performed by bi-linear interpolation. FSLEs are obtained for the points **r** of a lattice with spacing *δ*_0_ = *δ*. Initial conditions for which the prescribed final separation *δ*_*f*_ = r*δ* has not been reached even when using all available times in the dataset are assigned a value *λ* = 0.

### Finite Domain Lagrangian Divergence

As a complementary transport diagnosis we develop another Lagrangian quantity related to the geometric properties of the divergence of the velocity field. Three dimensional incompressible flows are non-divergent, and deviations in the horizontal divergence (${\nabla }_{H}\cdot {\bf{v}}$) are compensated with variations of the vertical velocity, w, *i.e*.,

4 $${\nabla }_{H}\cdot {\bf{v}}=\frac{\partial u(x,y)}{\partial x}+\frac{\partial v(x,y)}{\partial y}=-\frac{\partial w}{\partial z},$$

where u and v are the zonal and meridional components of the HF radar velocity fields. The divergence is the amount of flux entering or leaving a point. It can be thought as the rate of flux expansion (positive divergence) or the flux contraction (negative divergence). The flow expansion/contraction at the sea surface can be related to upwards/downwards vertical velocities. Since the HFR only measures the first meter of the ocean surface layer, the velocity field given by the HFR corresponds to the top layer where vertical velocities are equal to zero ($w(z=\mathrm{0)}=\mathrm{0)}$. Thus, we deduced from Eq. 4 that divergence at the top of the ocean overcomes upward motions, while upper ocean convergence zones overcome sinking flow. A positive horizontal divergence (negative divergence) of this volume flux would then drive upwelling (downwelling) at the top of the Ekman layer.

Assuming that HFR velocity field is divergent, we accumulate the instantaneous horizontal divergence along a trajectory $s({x}_{0},{y}_{0},{t}_{0})$ in the limited domain, which we refer to as Finite-Domain Lagrangian Divergence, integrating and averaging its value over time as followed:

5 $$FDLD({x}_{0},{y}_{0},{t}_{0},{t}_{f})=\frac{1}{T}{\int }_{{t}_{0}}^{{t}_{f}}{\nabla }_{H}\cdot {\bf{v}}(x(t),y(t),t)dt,$$

where $T={t}_{f}-{t}_{0}$, is the time interval of the trajectory integration. As for the FSLE, we define the Finite-Domain Lagrangian Divergence (FDLD) as the integrated Eulerian divergence of the fluid particles over their trajectories within the finite area covered by the HFR. Note that in our computations *t*_*f*_ may be different for each particle. All particles are integrated during a fixed time interval *T*, whose value must be chosen depending on the process studied and on the available domain (covered by the velocity field). To prevent trajectories to escape area during the integration, *T* must be lower or equal to the residence time (*RT*) in the region of interest. In our case, we use *T* = 5 days (typical response time of phytoplankton bloom in coastal fronts) when possible and $T=RT$ otherwise, with *RT* ranging from 3 to 8 days in our region of interest. This Lagrangian measure has recently been applied in atmospheric flows by Tang *et al*.^47^: based on LiDAR observations, they compared the LCSs against the Lagrangian Divergence ridges, as a proxy of divergent and convergent regions, to finally interpret the 3D structures of the flow. We also refer the readers to Huntley *et al*.^49^, who performed an extensive comparison between Finite-Time Lyapunov Exponents (FTLE) and Lagrangian Divergence using modeled data. They proved that FTLE can be expressed as the sum of the stretch and dilation rates, with the latter being the averaged divergence along the trajectories of the fluid particles. A rough approximation of the relationship between classical Lyapunov Exponents and Lagrangian divergence is given in Section S3 in the Supplementary Information following Falkovich *et al*.^68^. Here we use HFR velocities, which represent the total velocity at the sea surface, and we compare these geometrical quantities with biological observations. In the case of the currents obtained from satellite altimetry the geostrophic approximation has to be considered. The geostrophic balance between horizontal pressure gradients in the ocean and the Coriolis force results in horizontal currents (zero vertical velocities) with a neglected horizontal divergence. Therefore the divergent/convergent effects cannot be observed by satellite altimetry.

## Electronic supplementary material

Supplementary Information

Supplementary movie SM2

Supplementary movie SM2

## References

[1] Bauer JE (2013). The changing carbon cycle of the coastal ocean. Nature.

[2] Cloern JE, Foster SQ, Kleckner AE (2014). Phytoplankton primary production in the world’s estuarine-coastal ecosystems. Biogeosciences.

[3] Pauly, D. *et al*. Fisheries in Large Marine Ecosystems: Descriptions and Diagnoses. In *The UNEP Large Marine Ecosystem Report: a Perspective on Changing Conditions in LMEs of the World Regional Seas. UNEP Regional Seas Reports and Studies* (eds. Sherman, K. and Hempel, G.) 23–40 (2008).

[4] Haidvogel D, Blanton J, Kindle J, Lynch D (2000). Coastal Ocean Modeling: Processes and Real-Time Systems. Oceanography.

[5] Orfila A (2015). Empirical forecasting of HF-radar velocity using genetic algorithms. IEEE Transactions on Geoscience and Remote Sensing.

[6] Martin A (2003). Phytoplankton patchiness: the role of lateral stirring and mixing. Progress in Oceanography.

[7] Mahadevan A (2016). Impact of submesoscale physics on primary productivity of plankton. Annual Review of Marine Science.

[8] Oschlies A (1998). & Garçon, V. Eddy-induced enhancement of primary productivity in a model of the North Atlantic Ocean. Nature.

[9] Lehahn Y, d’Ovidio F, Lévy M, Heyfetz E (2007). Stirring of the Northeast Atlantic spring bloom: A Lagrangian analysis based on multisatellite data. J. Geophys. Res..

[10] Tew Kai E (2009). Top marine predators track Lagrangian coherent structures. Proc. Natl. Acad. Sci. USA.

[11] Calil P, Richards K (2010). Transient upwelling hot spots in the oligotrophic North Pacific. J. Geophys. Res.

[12] Rossi V, López C, Sudre J, Hernández-Garca E (2008). & Garçon, V. Comparative study of mixing and biological activity of the Benguela and Canary upwelling systems. Geophys. Res. Lett..

[13] Hernández-Carrasco I, Rossi V, Hernández-Garca E, Garçon V, López C (2014). The reduction of plankton biomass induced by mesoscale stirring: A modeling study in the Benguela upwelling. Deep Sea Research Part I: Oceanographic Research Papers.

[14] Gruber N (2011). Eddy-induced reduction of biological production in eastern boundary upwelling systems. Nature Geoscience.

[15] Lumpkin R, Elipot S (2010). Surface drifter pair spreading in the North Atlantic. Journal of Geophysical Research: Oceans.

[16] Poje AC (2014). Submesoscale dispersion in the vicinity of the Deepwater Horizon spill. Proceedings of the National Academy of Sciences.

[17] Corrado R, Lacorata G, Paratella L, Santoleri R, Zambianchi E (2017). General characteristics of relative dispersion in the ocean. Sci. Rep..

[18] Poje AC, Haza AC, Özgökmen TM, Magaldi MG, Garraffo ZD (2010). Resolution dependent relative dispersion statistics in a hierarchy of ocean models. Ocean Modelling.

[19] Zhong Y, Bracco A (2013). Submesoscale impacts on horizontal and vertical transport in the Gulf of Mexico. Journal of Geophysical Research: Oceans.

[20] Haza A, Özgökmen T, Hogan P (2016). Impact of submesoscales on surface material distribution in a Gulf of Mexico mesoscale eddy. Ocean Modelling.

[21] Beron-Vera F, LaCasce J (2016). Statistics of Simulated and Observed Pair Separations in the Gulf of Mexico. Journal of Physical Oceanography.

[22] Choi J (2017). Submesoscale Dynamics in the Northern Gulf of Mexico. Part III: Lagrangian Implications. Journal of Physical Oceanography.

[23] Capet X, McWilliams JC, Molemaker MJ, Shchepetkin AF (2008). Mesoscale to Submesoscale Transition in the California Current System. Part I: Flow Structure, Eddy Flux, and Observational Tests. Journal of Physical Oceanography.

[24] Berta M, Griffa A, Özgökmen TM, Poje AC (2016). Submesoscale evolution of surface drifter triads in the Gulf of Mexico. Geophysical Research Letters.

[25] McWilliams, J. C. Submesoscale currents in the ocean. *Proceedings of the Royal Society of London A: Mathematical, Physical and Engineering Sciences* **472** (2016). 10.1098/rspa.2016.0117 PMC4893189 27279778

[26] Griffa A (2013). Investigating transport pathways in the ocean. Deep Sea Research Part II: Topical Studies in Oceanography.

[27] Paduan JD, Washburn L (2013). High-frequency radar observations of ocean surface currents. Ann. Rev. Mar. Sci..

[28] Bellomo L (2015). Toward an integrated HF radar network in the Mediterranean Sea to improve search and rescue and oil spill response: the TOSCA project experience. Journal of Operational Oceanography.

[29] Lana A, Marmain J, Fernández V, Tintoré J, Orfila A (2016). Wind influence on surface current variability in the Ibiza Channel from HF Radar. Ocean Dynamics.

[30] García-Lafuente J, López-Jurado J, Cano-Lucaya N, Vargas-Yanez M, Aguiar-García J (1995). Circulation of water masses through the Ibiza Channel. Oceanologica Acta.

[31] Heslop EE (2012). Autonomous underwater gliders monitoring variability at choke points in our ocean system: A case study in the Western Mediterranean Sea. Geophysical Research Letters.

[32] Sayol J-M (2013). Sea surface transport in the Western Mediterranean Sea: A Lagrangian perspective. Journal of Geophysical Research: Oceans.

[33] Jansá J, López-Jurado J, Morillas-Kieffer A, Amengual B (1998). Seasonal and mesoscale variability of biological and chemical parameters related to the hydrodynamics of the Ibiza Channel (western Mediterranean). Bol. Inst. Esp. Oceanogr.

[34] Héloïse L (2013). Enhancing the comprehension of mixed layer depth control on the Mediterranean phytoplankton phenology. Journal of Geophysical Research: Oceans.

[35] Haller G (2015). Lagrangian coherent structures. Annual Review of Fluid Mechanics.

[36] Aurell E, Boffetta G, Crisanti A, Paladin G, Vulpiani A (1997). Predictability in the large: an extension of the Lyapunov exponent. J. Phys. A.

[37] Boffetta G, Lacorata G, Redaelli G, Vulpiani A (2001). Detecting barriers to transport: A review of different techniques. Physica D.

[38] d’Ovidio F, Fernández V, Hernández-García E, López C (2004). Mixing structures in the Mediterranean sea from Finite-Size Lyapunov Exponents. Geophys. Res. Lett..

[39] Hernández-Carrasco I, López C, Hernández-García E, Turiel A (2011). How reliable are finite-size Lyapunov exponents for the assessment of ocean dynamics?. Ocean Modelling.

[40] Galan A, Orfila A, Simarro G, Hernández-Carrasco I, López C (2012). Wave mixing rise inferred from Lyapunov exponents. Environmental Fluid Mechanics.

[41] Huhn F (2012). Horizontal Lagrangian transport in a tidal-driven estuary - Transport barriers attached to prominent coastal boundaries. Continental Shelf Research..

[42] Hernández-Carrasco I, López C, Orfila A, Hernández-Garca E (2013). Lagrangian transport in a microtidal coastal area: the Bay of Palma, island of Mallorca, Spain. Nonlinear Processes in Geophysics.

[43] Lekien F (2005). Pollution release tied to invariant manifolds: A case study for the coast of Florida. Physica D..

[44] Gildor H, Fredj E, Steinbuck J, Monismith S (2009). Evidence for Submesoscale Barriers to Horizontal Mixing in the Ocean from Current Meauserements and Aerial Photographs. Journal of Physical Oceanography.

[45] Haza AC (2010). Transport properties in small-scale coastal flows: relative dispersion from VHF radar measurements in the Gulf of La Spezia. Ocean Dynamics.

[46] Berta M (2014). Surface transport in the Northeastern Adriatic Sea from FSLE analysis of HF radar measurements. Continental Shelf Research.

[47] Tang W, Chan PW, Haller G (2011). Lagrangian coherent structure analysis of terminal winds detected by lidar. part ii: Structure evolution and comparison with flight data. Journal of Applied Meteorology and Climatology.

[48] Kalda J, Soomere T, Giudici A (2014). On the finite-time compressibility of the surface currents in the Gulf of Finland, the Baltic Sea. Journal of Marine Systems.

[49] Huntley HS, Lipphardt BL, Jacobs G, Kirwan AD (2015). Clusters, deformation, and dilation: Diagnostics for material accumulation regions. Journal of Geophysical Research: Oceans.

[50] Jacobs GA (2016). Ocean processes underlying surface clustering. Journal of Geophysical Research: Oceans.

[51] Kalampokis A, Uttieri M, Poulain PM, Zambianchi E (2016). Validation of HF Radar-Derived Currents in the Gulf of Naples With Lagrangian Data. IEEE Geoscience and Remote Sensing Letters.

[52] Solabarrieta L (2016). Skill Assessment of HF Radar???Derived Products for Lagrangian Simulations in the Bay of Biscay. Journal of Atmospheric and Oceanic Technology.

[53] Boffetta G, Celani A, Cencini M, Lacorata G, Vulpiani A (2000). Non-asymptotic properties of transport and mixing. Chaos.

[54] Richardson LF (1926). Atmospheric diffusion shown on a distance-neighbour graph. Proceedings of the Royal Society of London A: Mathematical, Physical and Engineering Sciences.

[55] Frisch, U. *Turbulence: The Legacy of A.N. Kolgomogorv*. (Cambrige University Press, 1995).

[56] Schroeder K (2011). Relative dispersion in the Liguro-Provençal basin: From sub-mesoscale to mesoscale. Deep Sea Research Part I: Oceanographic Research Papers.

[57] Estrada M (2014). Seasonal and mesoscale variability of primary production in the deep winter-mixing region of the NW Mediterranean. Deep Sea Research Part I: Oceanographic Research Papers.

[58] Franks PJS (1992). Phytoplankto blooms at fronts: Patterns Scales, and physical forcings mechanisms. Reviews in Aquatic Sciences.

[59] Vilibić I (2016). Self-organizing maps-based ocean currents forecasting system. Scientific Reports.

[60] Kaplan DM, Lekien F (2007). Spatial interpolation and filtering of surface current data based on open-boundary modal analysis. Journal of Geophysical Research: Oceans.

[61] Rio M-H (2014). Computation of a new mean dynamic topography for the Mediterranean Sea from model outputs, altimeter measurements and oceanographic *in situ* data. Ocean Science.

[62] Artale V, Boffetta G, Celani A, Cencini M, Vulpiani A (1997). Dispersion of passive tracers in closed basins: Beyond the diffusion coefficient. Phys. Fluids.

[63] Lacorata G, Aurell E, Vulpiani A (2001). Drifter dispersion in the Adriatic sea: Lagrangian data and chaotic model. Ann. Geophysicae.

[64] Hernández-Carrasco I, López C, Hernández-Garca E, Turiel A (2012). Seasonal and regional characterization of horizontal stirring in the global ocean. J. Geophys. Res..

[65] Haza AC, Poje AC, Özgökmen TM, Martin P (2008). Relative dispersion from a high-resolution coastal model of the Adriatic Sea. Ocean Modell..

[66] Özgökmen TM (2012). On multi-scale dispersion under the influence of surface mixed mixed layer instabilities and deep flows. Ocean Modell..

[67] Shadden SC, Lekien F, Marsden JE (2005). Definition and properties of Lagrangian coherent structures from finite-time Lyapunov exponents in two-dimensional aperiodic flows. Physica D.

[68] Falkovich G, Gawedzki K, Vergassola M (2001). Particles and fields in fluid turbulence. Rev. Mod. Phys..

