# Supplementary material for: Effect of small scale transport processes on phytoplankton distribution in coastal seas
Source: Sci Rep. 2018 Jun 5;8:8613. doi: 10.1038/s41598-018-26857-9 (PMC5988812; doi:10.1038/s41598-018-26857-9)
Supplement: Supplementary file 1 — Supplementary Information [file 41598_2018_26857_MOESM1_ESM.pdf]

Supplementary Information for "Effect of small scale transport processes on phytoplankton distribution in coastal seas"

I. Hernández-Carrasco[1], A. Orfila[2], V. Rossi[3], and V. Garçon[4]

[1]Balearic Islands Coastal Observing System, ICTS-SOCIB, Parc Bit, Edificio Naorte, 2nd floor, 07121 Palma de Mallorca, Spain.
[2]Marine Technology and Operational Oceanography Department, IMEDEA (CSIC-UIB), Esporles, Spain.
[3]Mediterranean Institute of Oceanography, CNRS UMR 7294, Campus de Luminy, 13288 Marseille, France.
[4]LEGOS, Laboratoire d'Etudes en Géophysique et Océanographie Spatiales, 18, Avenue Edouard Belin, 31401 Toulouse Cedex 9, France

May 8, 2018

S1. Area of study

The Ibiza channel (Fig. S1) is located in the Western Mediterranean, between the Spanish coast and Ibiza (Balearic Islands). Figure S1 shows the location map of the study area including a sketch of the regional circulation.

S2. Lagrangian validation of trajectories and Lagrangian Coherent Structures derived from HF Radar velocity fields

A Lagrangian validation has been performed using 8 drifter available in the domain of interest (HFR area of coverage) during the period of study (July 2012 – July 2014), in order to provide evidence on the reliability of the trajectories and the LCS computations using the HF Radar. Following Kalampokis *et al.* 2016 [2], we first compute the distance (D) between the virtual drifter trajectories advected by the HFR velocity field and the 8 real drifter trajectories. The integration of virtual drifter trajectories is initialized at the same position of the real drifters and then the distance between both drifters each hour are computed, stopping at 24 hours of integration. We reinitialize the virtual drifter position at the same position of the real drifter every 1 h, instead of 24 h, following other authors [3]. Fig. S2 a) shows the values of the distance as a funtion of the time for every real drifter trajectory and Fig. S2 b) the mean separation distance averaging over all the trajectories. One can appreciate that largest separation distances have been found for drifters deployed in October 2012 and April 2013 reaching a separation distance of 7-8 km. The smallest separations have been found for the drifters deployed in September 2012, showing different dynamical conditions between both

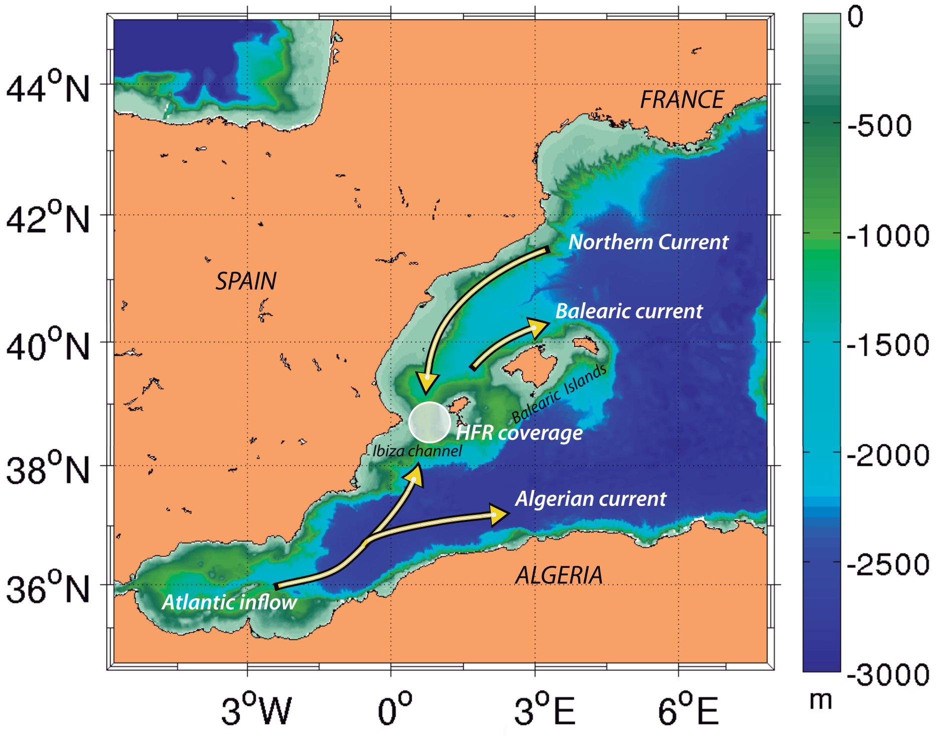

Figure S 1 – Map of western Mediterranean Sea showing the location and description of the local circulation in the Ibiza channel. The colorbar is the bathymetry in meters. The area of the HF Radar coverage is denoted by the white circle. The yellow arrows show the main currents.

months. The averaged distance of separation after 24 hours is about 4 km, which shows a good accuracy of the HF Radar measurements and in the trajectories computations.

Then we have validated the HFR-LCS computations comparing the position of the real drifters with the LCS. In Figure 1, of the manuscript we show the trajectory of a drifter during 60 hours in October 2012 in the area covered by the HFR together with the LCS showing the ability of such structures in determining the position of the drifter. Additionally, in the supplementary material we provided 2 videos showing the hourly evolution of the LCS from the FSLE together with drifters from 2 experiments performed on September 19-21 2012 and on April 16-17 2013. Although the purpose of this work is not to show the ability of the LCS in structuring the flow which has been already treated in several works, it can be observed how drifters follows the ridges defined by the HFR-LCS and thus this Lagrangian metric inferred from the finite domain of the HFR coverage is able to organize the dynamics of the area.

To evaluate the effect of the backward LCS to constrain the motion of the drifters we have compared the probability distribution function of the values of the HF Radar derived FSLE field at the position of the real drifter trajectories with the FSLE values for the whole HF Radar area (Fig. S2 c). In contrast with the low FSLEs found in the rest of the domain, a clear peak of high backward FSLE is found at the drifters locations; it shows that drifters are attracted to backward LCSs. The analysis shown here provides some evidences that the

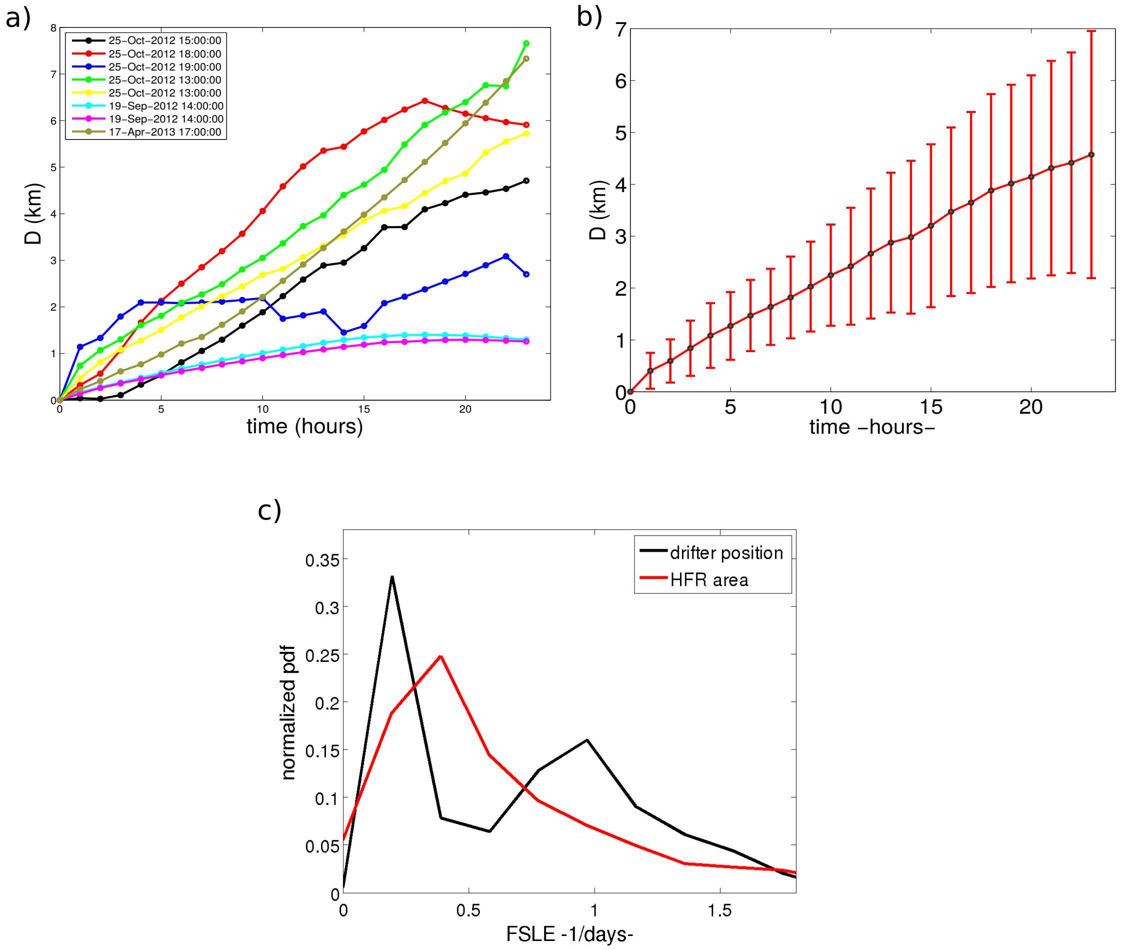

Figure S 2 – a) Distance of separation as a function of time, D(t), between real and synthetic drifters averaged over all the reinitializations for each real drifter. b) Distance of separation as a function of time, D(t), between real and synthetic drifters averaged over all the reinitializations and over all the drifters trajectories. c) Normalized probability distribution function of the values of FSLE at the drifters positions (black), and over the whole HF Radar domain at the same dates as the drifter trajectories (red).

HFR-derived LCS are reliable and can be used to identify dynamical structures in a finite area.

S3. Comparison between Lagrangian Divergence and Finite Size Lyapunov Exponents

Lyapunov exponents and Lagrangian divergence are related under geometrical considerations (see Falkovich *et al.* 2001 [1] for the details). We only show here a rough approximation of the relationship between both quantities taken from Falkovich *et al.* 2001 [1].

The separation Δ between two fluid particles trajectories $\mathbf{X}_1$ and $\mathbf{X}_2$, $\Delta_{12} = \mathbf{X}_1 - \mathbf{X}_2$, evolves satisfying the equation

$$\dot{\Delta} = \mathbf{V}(\mathbf{X}_1, t) - \mathbf{V}(\mathbf{X}_2, t). \tag{1}$$

For smooth velocities, we can approximate $\mathbf{V}(X_1, t) - \mathbf{V}(X_2, t) \approx \sigma(t)\Delta(t)$, where $\sigma_{ij}(t) = \nabla_j v^i(X(t), t)$, is the Lagrangian strain tensor, and thus,

$$\dot{\Delta}(t) = \sigma(t)\Delta(t), \tag{2}$$

leading to the linear evolution of the separation:

$$\Delta(t) = F(t)\Delta(0), \tag{3}$$

where F is the evolution matrix, defined as $F(t) = \partial R^i(x; t)/\partial x^j$.

In the case of a sea surface 2-D flow the area growth rate, L, tends to the Lagrangian average of the trace of the strain, $tr(\sigma) = \sum_{i=1}^{2} \sigma_{ii} = \nabla_H \cdot \mathbf{v}$, which corresponds to the horizontal Lagrangian divergence. It can be shown [1] that for short-correlated strain $det(F)^{-1}$ can be expanded as $1 - \int_0^t \langle \nabla_H \cdot \mathbf{v} \rangle \, dt$, and L given by $1/t \, \langle \ln \det[\mathrm{F(x,t)}] \rangle = \int_0^t \langle \nabla_H \cdot \mathbf{v}(x(t'), t') \rangle \, dt'$,

Let α_1 and α_2 be the eigenvalues of the 2D strain tensor F, we have that the growth rate of the area can be expressed by means of this two eigenvalues. Since the Lyapunov exponents, λ_L, can be expressed as

$$\lambda_{Li} = \frac{1}{t} ln|F\mathbf{f}_i|, \tag{4}$$

where $\mathbf{f}_i$ are the orthogonal eigenvectors of the directions of maximum deformation, associated to the eigenvalues α_i, we can obtain that the sum of the Lyapunov Exponents associated to this eigenvalues are related to the Lagrangian divergence by

$$\sum_{i=1}^{2} \lambda_{Li} = \frac{1}{t} \int_0^t \left\langle \nabla_H \cdot \mathbf{v}(x(t'), t') \right\rangle dt' \tag{5}$$

We compare in Figure S3 maps of FSLE and FDLD. It can be seen how minimum values of FDLD correspond to maximum values in the FSLE field.

S4. Comparison between LCS from Altimetry and HFR currents

In order to compare the LCSs obtained by altimetry with those from HF Radar velocity, we plot in Fig. S4 the values of FSLE for both data sets corresponding to the same date.

References

[1] Falkovich, G., K. Gawedzki, and M. Vergassola (2001), Particles and fields in fluid turbulence, *Rev. Mod. Phys.*, *73*, 913 – 975.

[2] Kalampokis, A., M. Uttieri, P. M. Poulain and E. Zambianchi (2016), Validation of HF Radar-Derived Currents in the Gulf of Naples With Lagrangian Data, *IEEE Geoscience and Remote Sensing Letters*, *13*, 1452–1456.

[3] Solabarrieta, L., S. Frolov, M. Cook, J. Paduan, A. Rubio, M. Gonzalez, J. Mader and G. Charria (2016), Skill Assessment of HF Radar–Derived Products for Lagrangian Simulations in the Bay of Biscay, *Journal of Atmospheric and Oceanic Technology*, *33*,12, 2585–2597.

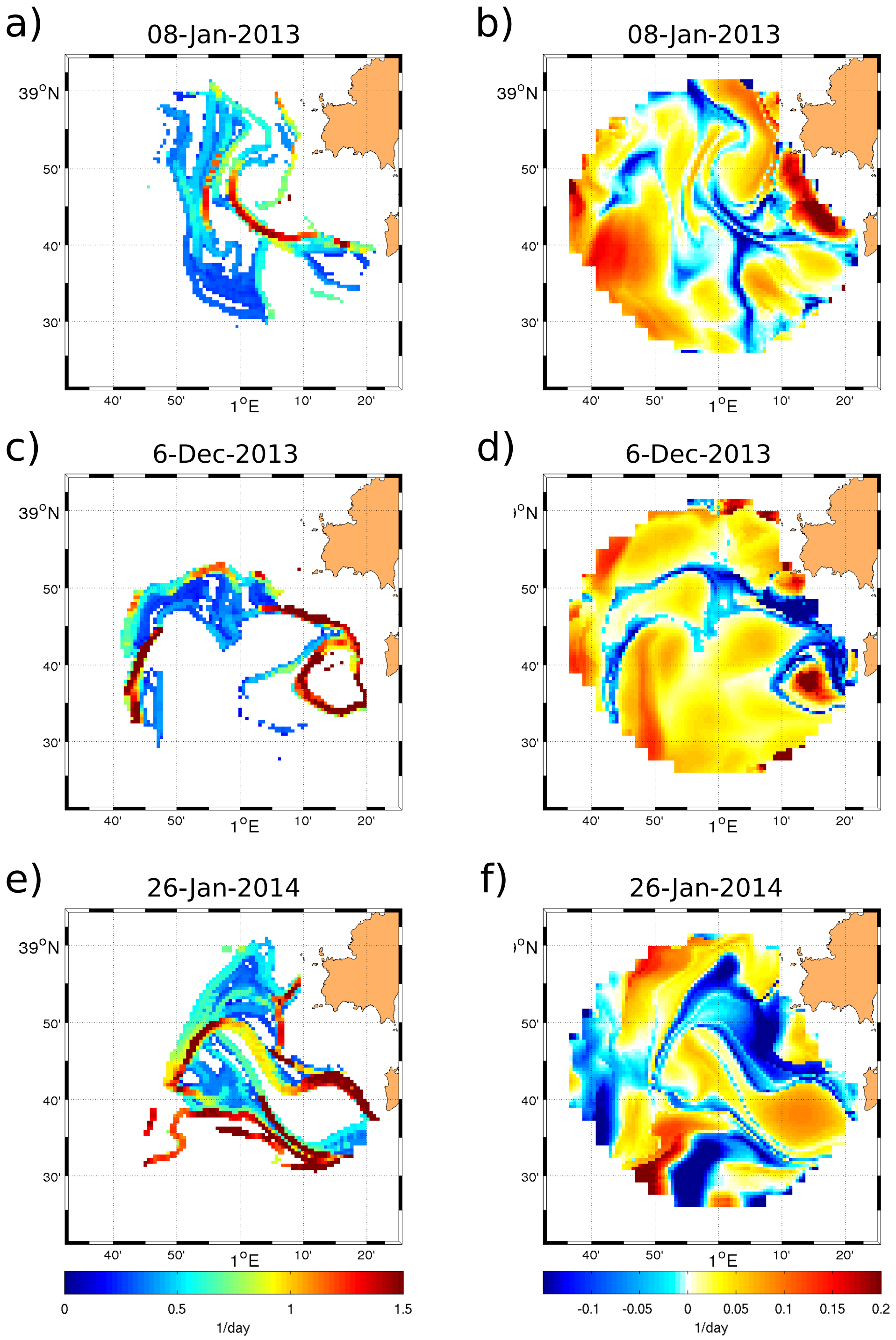

Figure S 3 – Left panel: Attracting LCSs corresponding to a) January 8th, 2013 (c) December 6th, 2013 (e) January 26th, 2013.Right panel: Snapshots of Lagrangian divergence (in days^{-1}) from HF Radar integrating 5 days trajectories of the particles computed for the same dates than the LCSs shown in left panels: (b) January 8th, 2013 (d) December 6th, 2013 (f) January 26th, 2014. The figure was made using MATLAB R2012a (http://www.mathworks.com).

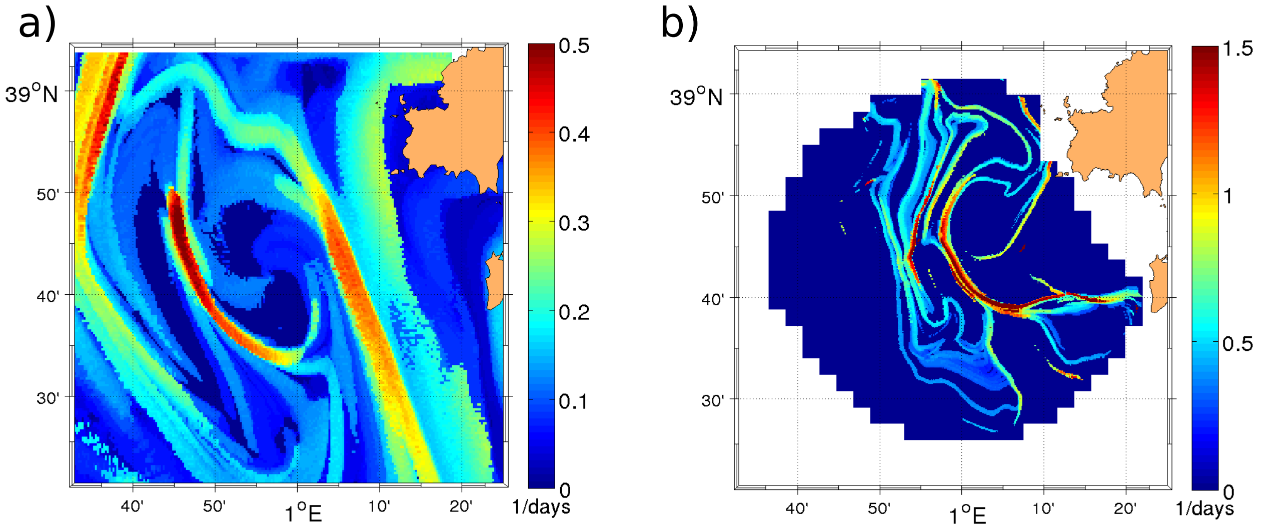

Figure S 4 – Maps of backward FSLEs corresponding to January 8, 2013 computed from a) altimetry and b) HF Radar in the Ibiza Channel using the same initial pair particle separation δ_0=400 m. The figure was made using MATLAB R2012a (http://www.mathworks.com).
